# Mass Spectrometry Analysis of Low-Molecular-Weight Sulfur-Containing Metabolites from an Infusion of *Scorodophloeus zenkeri* Stem Barks

**DOI:** 10.3390/molecules31214027

**Published:** 2026-11-09

**Authors:** Ericka Lorleil Mayindza Ekaghba, Simon Ollivier, Hélène Lavanant, Olivier Perruchon, Isabelle Schmitz, Carlos Afonso, Line Edwige Mengome, Corinne Loutelier-Bourhis, Patrice Lerouge

**Affiliations:** 1 Institut de Pharmacopée et Médecines Traditionnelles (IPHAMETRA), Centre National de la Recherche Scientifique et Technique (CENAREST), Libreville BP 12 141, Gabon; ericka.mayindza@iphametra.org (E.L.M.E.); line.mengome@iphametra.org (L.E.M.); 2 Université de Rouen Normandie (UNIROUEN), Normandie Univ, GlycoMEV UR 4358, SFR Normandie Végétal FED 4277, Innovation Chimie Carnot, 76000 Rouen, France; olivier.perruchon1@univ-rouen.fr; 3 Université de Rouen Normandie, INSA Rouen Normandie, Université de Caen Normandie, ENSICAEN, CNRS, Institut CARMeN UMR 6064, 76000 Rouen, France; simon.ollivier@univ-rouen.fr (S.O.); helene.lavanant@univ-rouen.fr (H.L.); isabelle.schmitz@cnrs.fr (I.S.); carlos.afonso@univ-rouen.fr (C.A.)

**Keywords:** mass spectrometry, phytochemical analysis, sulfur-containing molecules, *Scorodophloeus zenkeri*

## Abstract

*Scorodophloeus zenkeri* is a tree endemic to Central Africa that gives off a garlic-like odour. Therefore, its bark, seeds and wood are commonly used as a condiment in traditional foods in Cameroon and Gabon. Moreover, the infusion of the stem bark of *S. zenkeri* is used in traditional medicine in Gabon as a laxative. In the present study, we report on the phytochemical analysis by both ultra-high-performance liquid chromatography-tandem mass spectrometry and Fourier-transform ion cyclotron resonance mass spectrometry of the infusion of *S. zenkeri* bark. We focused our investigation in negative ion mode on sulfinic, sulfonic, sulfinosulfanyl, thiosulfuric acid derivatives and organosulfur metabolites containing up to four sulfur atoms. Rules for the MS/MS fragmentation of these unusual molecules are proposed.

## 1. Introduction

*Scorodophloeus zenkeri* Harms (Fabaceae) is a tree endemic to Central Africa. The infusion of its stem bark is widely used as a laxative in traditional Gabonese medicine [1]. Mixed with chilli peppers and bitter eggplants, it is also used to treat colds and coughs [2]. *S. zenkeri* is also renowned for its garlic-like odour and therefore, its bark, seeds and wood are commonly used as a condiment in traditional foods in Cameroon and Gabon. It is usually admitted that this garlic-like odour comes from sulfur-containing molecules that are produced, for instance, by garlic (*Allium sativum*) or onion (*Allium cepa*) [3,4]. Similar sulfur-containing molecules were also reported in the fungus *Marasmius alliaceus,* also known for its garlic-like odour [5]. The sulfur-containing molecules in these plants were identified as being mono-, di- and trisulfides, allyl isothiocyanate disulfide and S-allyl cysteine [3]. With regard to *S. zenkeri,* previous studies on the phytochemical analyses of hexane and dichloromethane extracts from their stem barks enabled the identification of organo-sulfur molecules that are composed of an alkane backbone with the insertion of sulfur atoms in carbon-carbon bonds. The main organo-sulfur compounds were metabolites ranging from trithiohexane to hexathioundecane [6] that have been previously identified in *Marasmius alliaceus* [5] or in alliaceous and cruciferous vegetables [7]. This indicates that the garlic-like odour of stem barks of *S. zenkeri* likely originates from its content in sulfur-containing molecules.

In the present study, we report on the phytochemical analysis of the infusion of *S. zenkeri* stem barks. We focused on the characterization of water-soluble sulfur-containing metabolites given their putative involvement in the garlic-like odour of this plant. Untargeted analyses, performed using both ultra-high-performance liquid chromatography coupled to high-resolution tandem mass spectrometry (UHPLC HRMS/MS) and Fourier-transform ion cyclotron resonance mass spectrometry (FTICR MS), allowed metabolite profiling and the description of their MS/MS fragmentation patterns in negative ion mode.

## 2. Results

The metabolites of stem bark of *Scorodophloeus zenkeri* were extracted by infusion in boiling water and then analyzed by UHPLC HRMS/MS in negative and positive ion modes after ultrafiltration of the infusion. A list of the main metabolites detected in the extract of *S. zenkeri* stem bark is reported in Table 1, Tables S1 and S2. However, we have focused this study on sulfur-containing metabolites, which account for a large number of molecules in this extract because these compounds are less frequently found in plants than terpenoids, phenolics, alkaloids, sugars, amino acids, lipids, etc. Furthermore, the literature reports little data concerning sulfur-containing metabolites. As these compounds are mostly acidic, they can easily deprotonate, yielding abundant [M−H]^−^ ions. Therefore, we focused the analysis on data obtained in the negative ion mode. From negative ion UHPLC HRMS/MS analysis using an Orbitrap instrument, 44 metabolites were detected, and putative annotations were proposed based on high-resolution accurate mass, MS/MS fragmentation patterns, and comparison with literature and/or databases (HMDB, MassBank, PubChem, GNPS Databases). This allowed the assignment of Level 2a/b to Level 3 identification for these uncommon metabolites according to [8] (Table 1). However, most of these metabolites are not recorded in spectral databases, nor described in the literature. Thus, we could only propose putative chemical structures based on MS/MS fragmentation patterns, being aware that several isomeric structures could be possible for some of them. Analysis by UHPLC HRMS/MS also enabled the separation of putative isomers. Since sulfur-containing molecules are referred to by various names in the literature, we have opted for the most commonly used nomenclature for metabolites investigated in this study. Their IUPAC nomenclature (https://chemsketch.fr.softonic.com) is presented in Table S3.

The first nine chromatographic peaks, between Rt = 0.55 and 1.60 min of the base peak chromatogram (BPC) (Figure S1), correspond to highly polar small sulfated (R-OSO_3_H), sulfinate (R-SO_2_H), sulfonate (R-SO_3_H), sulfinosulfanyl (R-S-SO_2_H) and S-alkyl thiosulfate derivatives (R-S-SO_3_H) (Table 1). The MS/MS spectra from their deprotonated molecules display diagnostic fragment ions of sulfate, sulfonate, alkylthiosulfate and sulfinate species, such as HSO_4_^−^(*m*/*z* 96.9601), HSO_3_^−^(*m*/*z* 80.9651), SO_3_^−^(*m*/*z* 79.9573), S_2_O_2_^−^(*m*/*z* 95.9345) and SO_2_^−^(*m*/*z* 63.9624) (Figure 1). Less polar organosulfur (R-S-R) metabolites were detected between Rt = 3.50 and 6.10 min and were characterized by detection of CH_3_S^−^ (*m*/*z* 46.9961) and C_2_H_5_S_2_^−^(*m*/*z* 92.9838) fragment ions. All these sulfur-containing fragment ions, as well as their fragmentation patterns, have been observed for different metabolites of the same chemical class, sharing the same chemical motifs. As an illustration, the HRMS/MS spectra of the [M−H]^−^ ion of ethane sulfinic acid show the SO_2_^−^ fragment ion at *m*/*z* 63.9628 (Figure 2A), and the HRMS/MS spectra of the [M−H]^−^ ion of a sulfinosulfanyl derivative exhibit the S_2_O_2_^−^ fragment ion at *m*/*z* 95.9343 (Figure 2B). With regard to sulfonic acids, fragmentation of their [M−H]^−^ ions is also characterized by the diagnostic fragment ion SO_3_^−^ at *m*/*z* 79.9573 (Figure 1C and Figure 2C, Table 1) in accordance with the MS^2^ fragmentation pattern of a standard methylsulfonic acid (HMDB0240280). When this acidic function is linked to an additional sulfur atom, two main fragment ions arise from [M−H]^−^ of the resulting thiopropane sulfonic acid derivatives and are detected at *m*/*z* 79.9573 and *m*/*z* 80.9651, corresponding to SO_3_^−^ and HSO_3_^−^ ions, respectively (Figure 1C and Figure 2D, Table 1). In addition, the SO_2_^−^ fragment ion at *m*/*z* 63.9624 is also observed (Figure 1C and Figure 2D, Table 1). This MS/MS fragmentation is in accordance with data reported on S-propyl thiosulfuric acid isolated from onion (HMDB0041066) and cysteine S-sulfate (HMDB0000731).

Other diagnostic fragment ions observed in negative UHPLC HRMS/MS were used for the annotation of organosulfur molecules, such as fragment ions at *m*/*z* 46.9961 (CH_3_-S^−^) and *m*/*z* 92.9838 (C_2_H_5_-S_2_^−^) resulting from the cleavage of the C-S bond of organosulfur backbones (Figure 1D and Figure 2E,F, Table 1). However, the *m*/*z* 92.9838 would more probably correspond to CH_3_-S-CH_2_-S^−^ ion as the MS/MS spectra of metabolites containing organosulfur backbone and carboxylic acid groups (Figure 2E,F) showed the occurrence of the fragment ion at *m*/*z* 92.9838 only in the HRMS/MS spectrum of the [M−H]^−^ ion of the metabolite exhibiting sulfur atoms at the 4 and 6 positions on the backbone (Figure 2F) and not in the homologue metabolite with a disulfide at positions 4 and 5 (Figure 2E). It should be noted that in the MS/MS spectra of these two carboxylic acids, [M –H − CO_2_]^−^ ions result from the loss of CO_2_ from [M−H]^−^ ions and are specific to the fragmentation of carboxylic acids in negative ion mode (Table 1). However, the position of the hydroxyl group on C2 or C3 of the acidic chain remains uncertain.

It is worth noting that the diagnostic negative fragment ions represented in Figure 1 and assigned to sulfur-containing molecules are also observed in larger metabolites exhibiting both organosulfur and acidic motifs. This enables the structural identification of metabolites having multiple sulfur atoms inserted in different chemical groups. This can be illustrated by the UHPLC HRMS/MS spectra of sulfinosulfanyl and S-alkyl thiosulfuric acid derivatives, as well as sulfur-containing diacid derivatives (Figure 3). The fragment ion at *m*/*z* 92.9838 arises from the cleavage of C-S bonds of sulfide groups (R-S-R). Other negative fragment ions were characteristic of sulfinosulfanyl derivatives (R-S-SO_2_H) with fragment ion S_2_O_2_^−^ at *m*/*z* 95.9345, of S-alkylthiosulfate (R-S-SO_3_H) with both fragment ions SO_3_^−^ at *m*/*z* 79.9573 and HSO_3_^−^ at *m*/*z* 80.9651 and sulfinate (R-SO_2_H) with fragment ion SO_2_^−^ at *m*/*z* 63.9624.

To validate all these annotations, given that these metabolites contain many sulfur and oxygen atoms, which could lead to various close isobaric combinations, the extract was investigated by ultra-high-resolution mass spectrometry using an 18-T FTICR MS, which enabled the assignment of a unique and reliable molecular formula for each deprotonated molecule, thus avoiding any misinterpretation. Moreover, this instrument offers exceptional mass resolution (m/Δm ≈ 10^6^) and accuracy (below 0.5 ppm), allowing the study of isotopic fine structures and isotopologue detection. This allows the elemental composition to be validated when experimental and simulated isotopic profiles match both in terms of exact *m*/*z* and relative abundances for each isotopologue ion. In our case, sulfur-containing compounds show characteristic isotopic profiles linked to the natural abundance of the ^32^S (95.0%), ^33^S (0.8%) and ^34^S (4.2%) isotopes, as well as those of ^12^C (98.9%) and ^13^C (1.1%) isotopes. This is illustrated in Figure S2 for the isotopic peaks associated with the monoisotopic ion at *m*/*z* 140.96855 (C_2_H_5_O_3_S_2_^–^), which showed an isotopic fine structure where the contributions of ^13^C, ^33^S and ^34^S could be clearly distinguished. The relative intensities confirmed the presence of two S atoms. Because of the 10^6^ mass resolution, the extract could be directly infused into the ESI source, and the deprotonated molecules were clearly detected and their molecular formula determined, confirming all the assignments reported in Table 1 and Table S2.

Other metabolites, more apolar, including aromatic acids and fatty acids, were also detected (Rt between 3.88 and 8.35 min) and annotated (Table S1) using databases (HMDB, MassBank, PubChem, GNPS Databases). Among these metabolites, hydroxy diacids (C7, C9 and C10) and hydroxy stearic acids were putatively identified using literature data on hydroxylated fatty acids (Table S1) [9,10,11]. These compounds likely derive from the degradation of suberin contained in barks [12]. In addition, the [M−H]^−^ ion at *m*/*z* 517.3166 could correspond to a triterpenoid (C_30_H_46_O_7_) exhibiting two carboxylic groups. However, the MS/MS spectrum did not allow the detailed identification of its structure (Table S1). Then, sulfated polyhydroxy C18 mono- and diacids were characterized by the occurrence in their MS/MS spectra of an intense ion at *m*/*z* 96.9601 assigned to the HSO_4_^−^ fragment ion (Figure 1A). Due to the lack of informative fragment ions, the structures of these fatty acids were not fully resolved, but they also likely derive from the oxidation of C18 mono- and diacids of suberin [12] (Table S2).

## 3. Discussion-Conclusions

The investigation by ultra-high-performance liquid chromatography-tandem mass spectrometry and by Fourier-transform ion cyclotron resonance mass spectrometry of the infusion of *S. zenkeri* bark enabled a tentative annotation in negative ion mode of unusual sulfur-containing metabolites containing up to four sulfur atoms. We focused on these metabolites because most of them are not structurally annotated using databases or reported in the literature. Therefore, it would be of interest to apply such an analytical strategy to other plants exhibiting a garlic-like odour, such as alliaceous and cruciferous vegetables, taking advantage of diagnostic reporter MS^2^ fragment ions reported in this study. It is worth noting that the origin of most oxidized sulfur-containing metabolites investigated in this article is questionable because we cannot exclude that some of these compounds were generated by oxidation of thiols or thioethers during the extraction process.

One of the main limitations of this study is the low availability of standard sulfur-containing molecules and therefore the difficulty of comparing either LC retention times or MS^2^ fragmentation patterns with molecules of known structure. Indeed, among metabolites reported in Table 1, only methylsulfonic acid, sulfoacetic acid and cysteine S-sulfate are commercially available and their MS data are reported in public databases (HMDB0240280, HMDB0041066 and HMDB0000731, respectively). Other metabolites have not been reported in the literature. Although comparison with standard molecules is lacking, the consistency of fragmentation patterns observed in metabolites sharing the same chemical motifs allows us to have confidence in their annotations. However, we cannot rule out that the differentiation between isomers was not fully resolved in this study, as noted in the results section for hydroxy carboxylic acids.

Regarding the mass spectrometers used in this study, FTICR MS was mainly used to validate the annotation of diagnostic deprotonated molecules by studying the isotopic fine structure, i.e., by comparing the exact *m*/*z* and abundances of isotopologues. Their MS^2^ fragmentation patterns and Rt were determined by UHPLC-HRMS/MS. These diagnostic reporter MS^2^ fragment ions will be very useful for the identification of molecules belonging to these chemical families, regardless of their origin, even when using more widely available mass spectrometers.

## 4. Materials and Methods

### 4.1. Plant Material

The harvesting of stem barks of *Scorodophloeus zenkeri* Harms (Fabaceae) was carried out in 2020 in Libreville, Gabon. A sample of this plant was deposited at the National Herbarium of Gabon, where it was identified and preserved. The stem bark was dried for two weeks at the Department of Traditional Medicine of the Institut de Pharmacopée et Médecines Traditionnelles (IPHAMETRA), Libreville, Gabon, and then reduced to a fine powder using a grinder. Five hundred grams of ground material were put in boiling water and then stirred for 15 min without any precaution to preserve the infusion from oxidation by atmospheric oxygen. The suspension was then filtered and the solution was allowed to cool to room temperature, and then frozen and freeze-dried to give the infusion of stem bark of *Scorodophloeus zenkeri*. The experiment was performed in triplicate. The yields of the infusion extracts were 1.8 ± 0.3% of the bark material.

### 4.2. Ultra-High Performance Liquid Chromatography-Tandem Mass Spectrometry (UHPLC HRMS/MS) Analysis

Five mg of the infusion of stem bark of *Scorodophloeus zenkeri* were dissolved in 1 mL of HPLC-grade water and then filtered through 0.5 mL centrifugal filters (Ultracel 10 kDa, Amicon, Miami, FL, USA) to remove high molecular weight compounds and impurities. Ultra-high-performance liquid chromatography coupled to tandem mass spectrometry (UHPLC HRMS/MS) analysis was carried out using an UHPLC system (Vanquish, Thermo Scientific, San Jose, CA, USA) coupled to a quadrupole-Orbitrap mass spectrometer (Exploris 120, Thermo Scientific, Bremen, Germany). The chromatographic separations were performed using a C18 column (Acquity UPLC HSS T3 1.8 µm × 2.1 mm × 100 mm, Waters Corporation, Milford, MA, USA) with a prefilter of 0.2 µm, kept at 50 °C during the analysis. An autosampler kept the samples at 6 °C. The injection volume was 3 µL. The solvents used for gradient separation were 0.1% (*v*/*v*) formic acid in water as mobile phase A and 0.1% (*v*/*v*) formic acid in acetonitrile as mobile phase B. The flow rate was 0.4 mL/min. The elution gradient was first 1% B for 1 min, then increased linearly to 100% B over 20 min and then maintained at 100% B for 8 min. Samples were analyzed in both negative and positive ion modes. The ESI source parameters were as follows: spray voltage 3500 V and 3000 V for positive and negative ion modes, respectively; sheath gas 35 (arbitrary units), auxiliary gas 10 (arbitrary units), sweep gas 2 (arbitrary units), ion transfer tube 320 °C and vaporizer temperature 275 °C. Data-dependent acquisitions were carried out in both positive and negative ion modes. MS1 resolution was set at 60,000 with a standard automatic gain control (AGC) target, a maximum injection time set to auto, a microscan to 1, RF lens to 70%, and a scan range from *m*/*z* 80 to 1200. EASY-IC internal standard (fluoranthene) was used. For MS/MS, resolution was set at 15,000 with a maximum injection time of 50 ms. The isolation window was 2 *m*/*z*, dynamic exclusion was set at 4 s, mass tolerance was ±4 ppm and the precursor intensity threshold was set at 5.10^5^ in positive mode and 1.10^5^ in negative mode. The normalized collision energies were 15%, 40% and 60% in both positive and negative ion modes. Data processing was carried out using MZmine 4.5 (version 4.5.0 [13,14]). Annotation was performed based on accurate mass measurements and HRMS/MS spectra according to the literature and databases.

### 4.3. Fourier-Transform Ion Cyclotron Resonance Mass Spectrometry (FT-ICR MS) Analysis

The filtered sample was diluted 100-fold in a 50/50 (*v*/*v*) water/methanol mixture (LC-MS grade, Carlo-Erba, Val de Reuil, France), and was then analyzed by direct infusion on a prototype timsMRMS instrument equipped with an 18-T superconducting magnet (Bruker, Bremen, Germany) [15]. Ionization was performed using an electrospray ion source at a flow rate of 120 µL/h, capillary voltage of 4.5 kV, drying gas temperature of 200 °C and drying gas flow rate of 4 L/min. Mass spectra were acquired using quadrupolar (2ω) detection over a mass range of *m*/*z* 80–700 with a data size of 8M points (transient signal of 0.5592 s). Key ion transmission in the mass spectrometer were funnels 1 and 2 RF amplitude 150 V_p-p_, multipole RF amplitude 150 V_p-p_, quadrupole filter low mass set to *m*/*z* 80, collision cell RF amplitude 500 V_p-p_. The transfer parameters to the ICR cell were: time-of-flight 0.6 ms, transfer hexapole RF frequency 6 MHz and amplitude 400 V_p-p_. To obtain fine isotopic structures, 500 scans were summed, each following a 300 ms accumulation in the collision cell of the instrument.

## Figures and Tables

**Figure 1:**
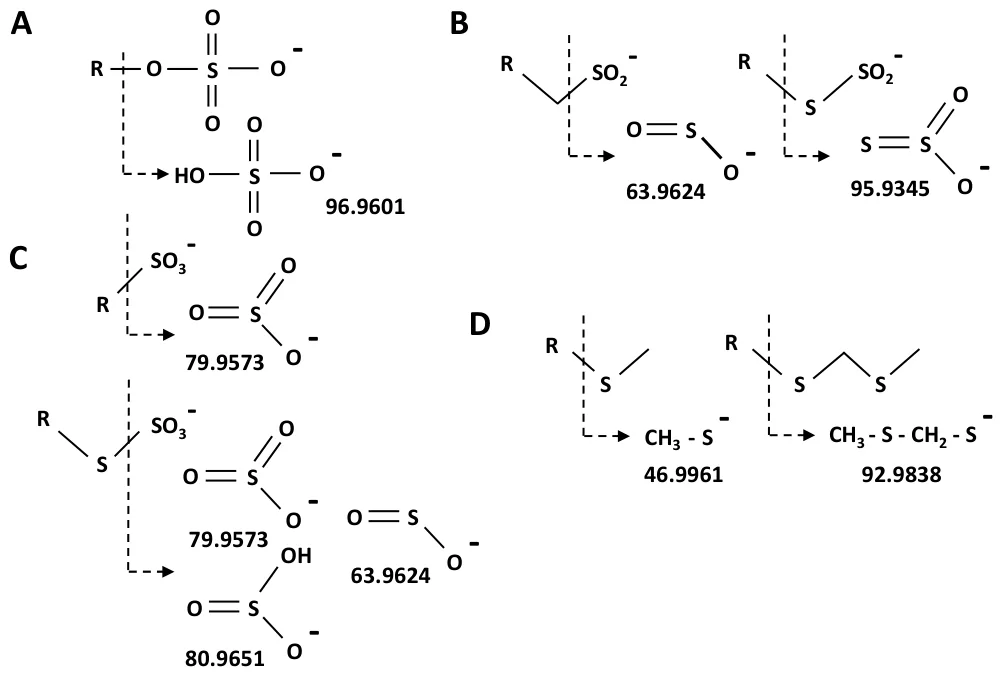
Diagnostic HRMS/MS negative fragment ions of (**A**) sulfated metabolites (R-SO_3_H), (**B**) sulfinic acid (R-SO_2_H) and sulfinosulfanyl derivatives R-S-SO_2_H), (**C**) sulfonic acid (R-SO_3_H) and S-alkyl thiosulfuric acid (R-S-SO_3_H) and (**D**) organosulfur (R-S-R) metabolites.

**Figure 2:**
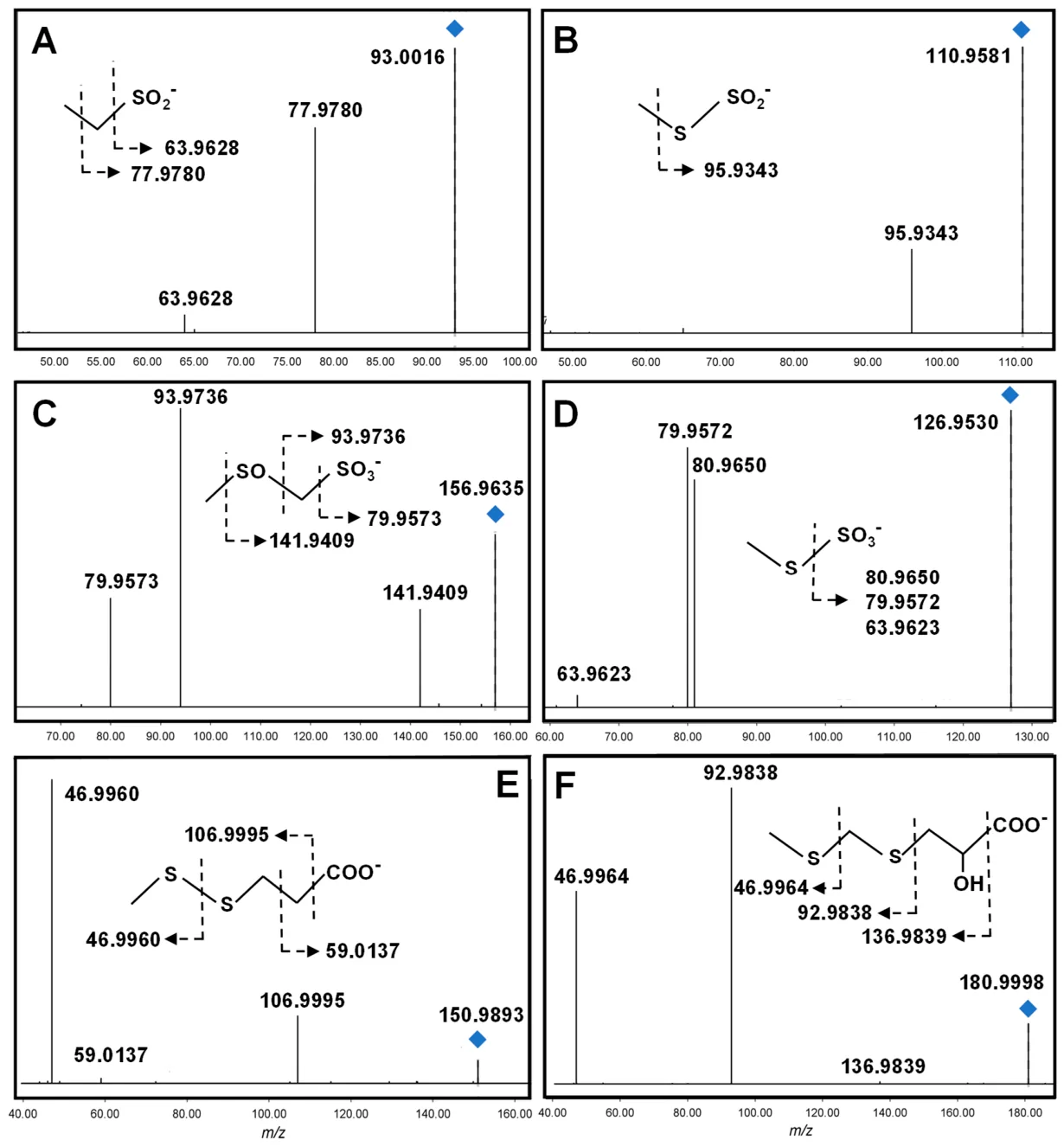
UHPLC HRMS/MS spectra of [M−H]^−^ ions of (**A**) ethane sulfinic acid at *m*/*z* 93.0016, (**B**) Sulfinosulfanyl methane at *m*/*z* 110.9581, (**C**) 2-oxo 2-thiopropane sulfonic acid at *m*/*z* 156.9635, (**D**) S-methyl thiosulfuric acid at *m*/*z* 126.9530, (**E**) 4,5-dithio hexanoic acid at *m*/*z* 150.9893 and (**F**) 2-hydroxy 4,6-dithio heptanoic acid at *m*/*z* 180.9998. See Figure 1 for the structure of sulfur-containing fragment ions detected in negative HRMS/MS. Diamond: precursor ion.

**Figure 3:**
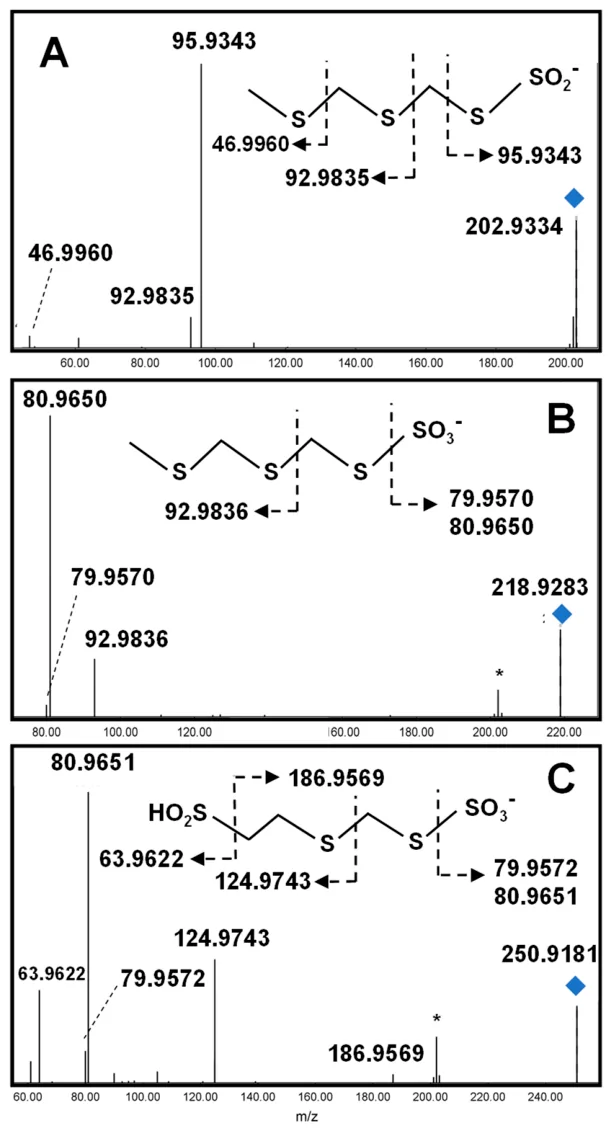
UHPLC HRMS/MS spectra of [M−H]^−^ ions of (**A**) Sulfinosulfanyl 2,4 dithiopentane at *m*/*z* 202.9334, (**B**) S-(2,4-dithiopentane) thiosulfuric acid at *m*/*z* 218.9283 and (**C**) S-(2-thiobutane-4-sulfinyl) thiosulfuric acid at *m*/*z* 250.9181. See Figure 1 for the structure of sulfur-containing fragment ions detected in negative HRMS/MS. Diamond: precursor ion. *: contamination ion.

**Table 1:** Proposed structures deduced from FTICR MS and UPLC HRMS/MS analyses in negative ion mode of sulfur-containing metabolites from the infusion of stem barks of *S. zenkeri*. Exp: experimental value determined by UHPLC-HRMS or FTICR MS. Calc: calculated value. * Underlined ions are represented in Figure 1. n.d.: not determined. ** Level identification according to [8].

Rt (min)	[M−H]^−^ (Mass Error in ppm)			Formula	MS/MS * Orbitrap	Putative Annotation		Level **
	Exp. Orbitrap	Exp. FTICR	Calc.			Name	Proposed Structure	
0.72	93.0016	n.d.	93.00157	C_2_H_6_O_2_S	63.96/ 77.98	Ethane sulfinic acid	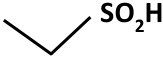	**2b**
1.27	124.9737	124.97366 (0.008)	124.97365	C_2_H_6_O_2_S_2_	63.96	1 or 2-Sulfanylethane sulfinic acid	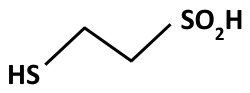	**3**
0.75	110.9581	110.95802 (0.182)	110.95800	CH_4_O_2_S_2_	46.99/95.93	Sulfino- sulfanyl methane	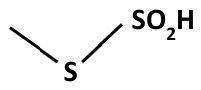	**2b**
1.59	156.9458	156.94572 (0.000)	156.94572	C_2_H_6_O_2_S_3_	46.99/92.98/95.93/ 110.96	Sulfinosulfanyl 2-thiopropane	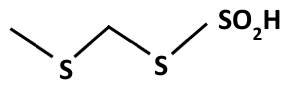	**2b**
4.40	202.9334	202.93340 (−0.20)	202.93344	C_3_H_8_O_2_S_4_	46.99/92.98/ 95.93/ 110.96	Sulfinosulfanyl 2,4 dithiopentane	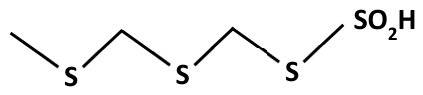	**3**
1.31	168.9999	168.99984 (−0.12)	168.99986	C_4_H_10_O_3_S_2_	62.99/95.93 /105.03	Sulfino-sulfanyl 1-hydroxybutane	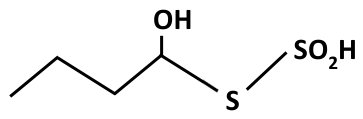	**3**
0.65	94.9808	94.98086 (0.21)	94.98084	CH_4_O_3_S	79.95	Methane sufonic acid	CH_3_-SO_3_H	**2a**
0.62	138.9707	138.97068 (0.07)	138.97067	C_2_H_4_O_5_S	79.95/ 94.98	Sulfoacetic acid	HOOC-CH_2_-SO_3_H	**2b**
0.94	140.9685	140.96855 (−0.07)	140.96856	C_2_H_6_O_3_S_2_	46.99/ 60.97/ 79.95	2-thiopropane sulfonic acid	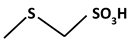	**2b**
0.67	156.9635	156.96350 (0.19)	156.96347	C_2_H_6_O_4_S_2_	79.95 /93.97 /141.94	2-oxo 2-thiopropane sulfonic acid	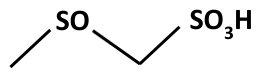	**2b**
0.97	184.9584	184.95840 (0.05)	184.95839	C_3_H_6_O_5_S_2_	79.95/ 140.06/166.95	4-sulfonyl 3-thiobutanoic acid	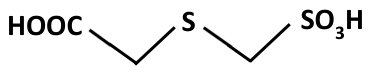	**3**
0.69	172.9584	172.95836 (−0.17)	172.95839	C_2_H_6_O_5_S_2_	63.96/79.95	2-sulphinyl ethanesulfonic acid	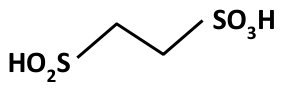	**2b**
0.86	126.9530	126.95289 (−0.16)	126.95291	CH_4_O_3_S_2_	46.99/79.95 80.96	S-methyl thio- sulfuric acid	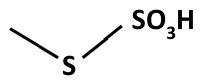	**2a**
0.59	199.9693	199.96924 (−0.25)	199.96929	C_3_H_7_NO_5_S_2_	79.95/80.96/ 112.94/120.13/136.00	Cysteine S-sulfate	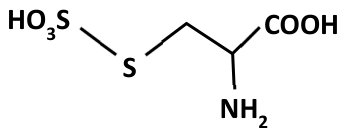	**2a**
5.18	218.9283	218.92835 (0.00)	218.92835	C_3_H_8_O_3_S_4_	79.95/80.96/92.98/ 126.95/138.97	S-(2,4 dithio pentane) thiosulfuric acid	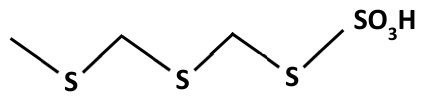	**2b**
3.54	2 50.9181	250.91813 (−0.20)	250.91818	C_3_H_8_O_5_S_4_	63.96/79.95/80.96/ 124.97	S-(2 thio butane 4-sulphinyl) thiosulfuric acid	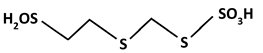	**3**
4.97	180.9998	180.99989 (0.17)	180.99986	C_5_H_10_O_3_S_2_	46.99/92.98 / 136.99	2-hydroxy 4,6-dithio heptanoic acid	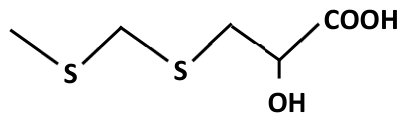	**3**
6.05	195.0155	195.01547 (−0.21)	195.01551	C_6_H_12_O_3_S_2_	46.99/92.98/ 101.02/ 149.03	2-hydroxy 5,7-dithio octanoic acid	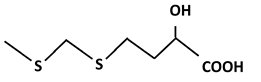	**3**
5.84	1 50.9893	150.98927 (−0.20)	150.98930	C_4_H_8_O_2_S_2_	46.99/ 59.01 /106.99	4,5-dithio hexanoic acid	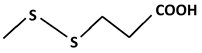	**2b**

## Data Availability

Data will be made available on request.

## Supplementary Materials

The supporting information can be downloaded at https://www.mdpi.com/article/10.3390/molecules31214027/s1. Figure S1. Base peak chromatogram for the UHPLC HRMS/MS in negative ion mode of the infusion of stem barks of *S. zenkeri*; Figure S2. (A) Zoom between *m*/*z* 140 and *m*/*z* 144.5 of the FTICR MS spectrum of [M−H]^−^ ion of 2-thiopropane sulfonic acid highlighting *m*/*z* values and relative ion intensities of ^33^S, ^34^S and ^13^C-containing metabolites. (B) Theorical spectrum of C_2_H_5_O_3_S_2_^−^; Table S1. Non sulfur-containing metabolites identified by UHPLC HRMS/MS in negative and positive ion modes in the infusion of *S. zenkeri* Exp: experimental value. Calc: calculated value; Table S2. Sulfated metabolites identified by UHPLC HRMS/MS in negative ion mode in the infusion of *S. zenkeri*. Exp: experimental value. Calc: calculated value; Table S3. Names of sulfur-containing metabolites.

## Abbreviations

The following abbreviations are used in this manuscript:

UHPLC HRMS/MS	Ultra-high-performance liquid chromatography-tandem mass spectrometry
FT-ICR MS	Fourier-transform ion cyclotron resonance mass spectrometry
