# Supplementary material for: Mass Spectrometry Analysis of Low-Molecular-Weight Sulfur-Containing Metabolites from an Infusion of Scorodophloeus zenkeri Stem Barks
Source: Molecules. 2026 Nov 9;31(21):4027. doi: 10.3390/molecules31214027 (PMC13764858; doi:10.3390/molecules31214027)
Supplement: Supplementary file 1 [file molecules-31-04027-s001.zip › molecules-4624409-supplementary.pdf]

Supplementary Material

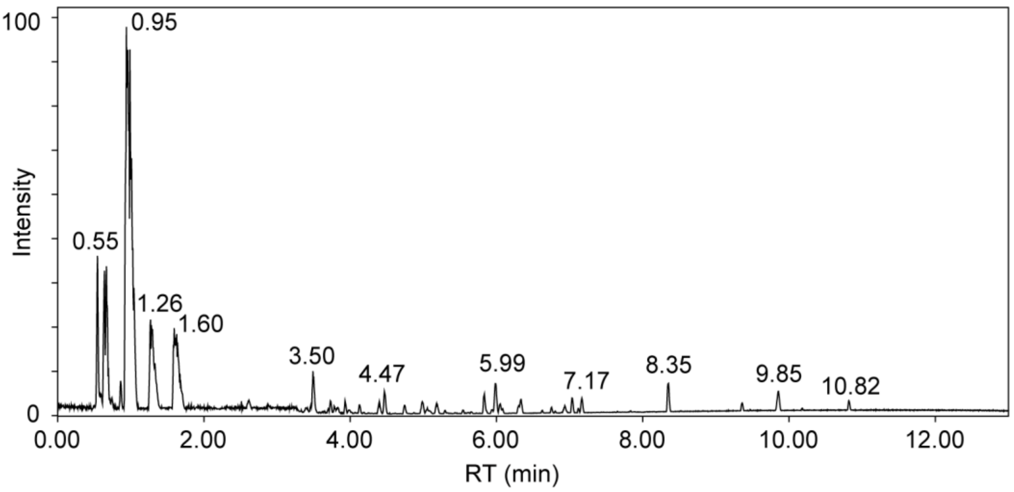

Figure S1. Base peak chromatogram for the UHPLC HRMS/MS in negative ion mode of the infusion of stem barks of *S. zenkeri*.

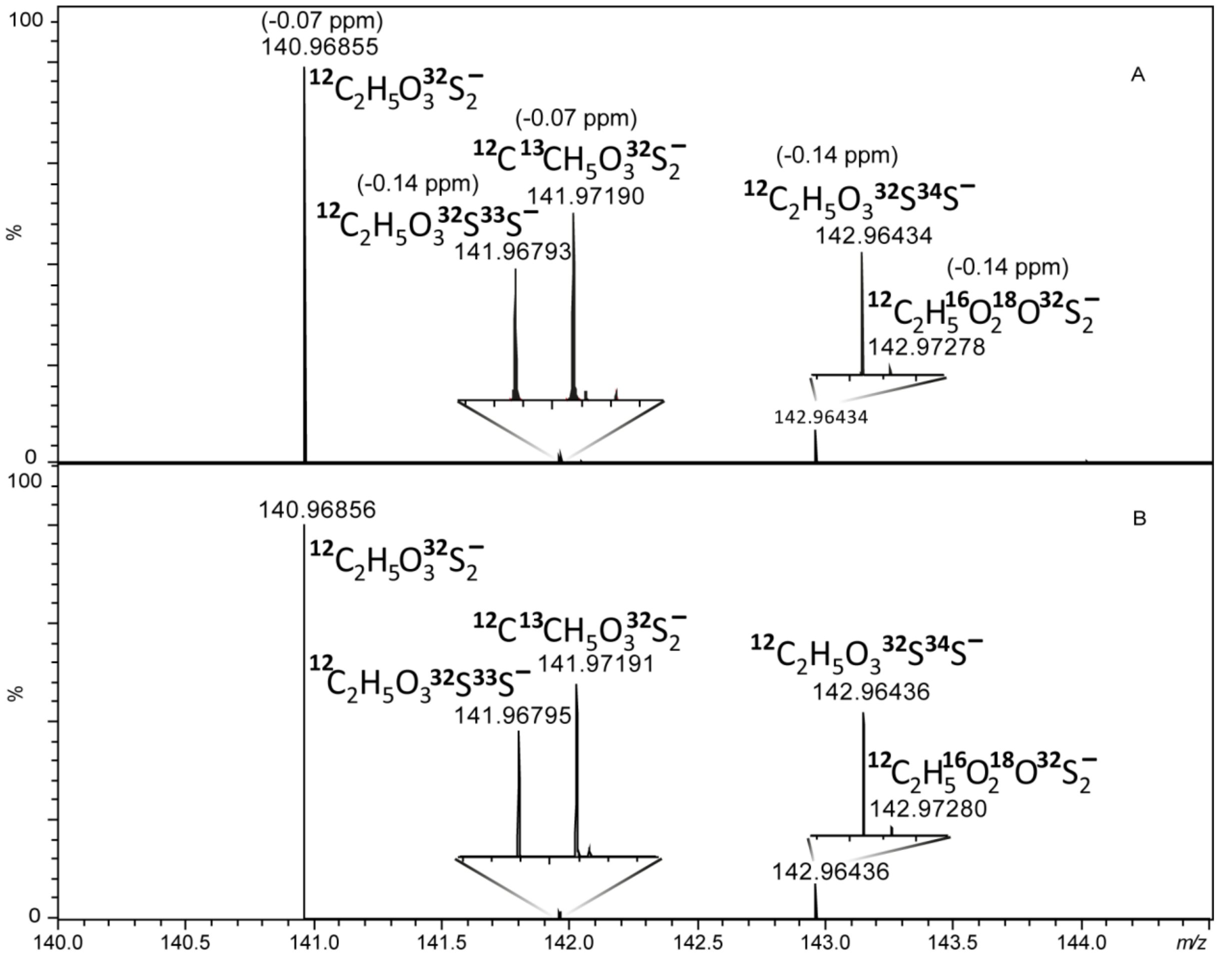

Figure S2. (A) Enlargement over the range *m/z* 140 to *m/z* 144.5 of the FTICR mass spectrum showing the isotopic pattern of $[M-H]^-$ ion of 2-thiopropane sulfonic acid. (B) Theorical spectrum of $C_2H_5O_3S_2^-$.

Table S1. Non sulfur-containing metabolites identified by UHPLC HRMS/MS in negative and positive ion modes in the infusion of *S. zenkeri* Exp: experimental value. Calc: calculated value.

RT	$[M-H]^-$		$[M+H]^{+\,a}$ $[M]^{+\,b}$		Molecular formula	Proposed metabolite	ESI MS/MS (-) or (+) ions (*m/z*)
	Exp m/z	*Calc m/z*	*Exp m/z*	*Calc m/z*			
0.61	146.0459	146.0459	148.0604[a]	148.0604[a]	$C_5H_9NO_4$	Glutamic acid	(+) 84/102/130
0.67			162.1124	162.1125	$C_7H_{16}NO_3$	Unidentified	(+) 58/72/116/134
0.69	135.0300	135.0298			$C_4H_8O_5$	Threonic acid	(-) 59/75
0.79			144.1019[b]	144.1019[b]	$C_7H_{14}NO_2$	Stachydrine	(+) 58/84
0.84	133.0142	133.0142			$C_4H_6O_5$	Malic acid	(-) 71/89/115
0.94			236.1493	236.1492	$C_{10}H_{21}NO_5$	Unidentified	(+) 87/131
0.95			118.0862[a]	118.0862[a]	$C_5H_{11}NO_2$	Valine	(+) 72
2.24			132.1019[a]	132.1018[a]	$C_6H_{13}NO_2$	Isoleucine/Leucine	(+) 86
3.56	164.0717	164.0717	166.0863[a]	166.0862[a]	$C_9H_{11}NO_2$	Phenylalanine	(+) 103/120
3.88	153.0191	153.0192			$C_7H_6O_4$	Dihydroxybenzoic acid	(-) 119
3.95	218.1033	218.1033			$C_9H_{17}NO_5$	Pantothenic acid	(-) 71/88/146
4.15	356.0984	356.0986			$C_{15}H_{19}NO_9$	Cyclodopaglucoside	(-) 115/179/ 194/312
4.45	233.1030	233.1030			$C_{10}H_{18}O_6$	Dihydroxydecanedioic acid	(-) 73/85/ 141/159/215
4.73	175.0612	175.0611			$C_7H_{12}O_5$	Hydroxy heptadioic acid	(-) 59/85/115/147
5.04	211.0247	211.0248			$C_9H_8O_6$	Carboxyvanillic acid	(-) 108/152/196
5.98	206.0821	206.0822			$C_{11}H_{13}O_3N$	N-acetylphenylalanine	(-) 58/91/147/164
6.09	217.1081	217.1081			$C_{10}H_{18}O_5$	Hydroxydecanedioic acid	(-) 73/97/125/ 143/155/199
6.33	245.0928	245.0931			$C_{13}H_{14}N_2O_3$	N-acetyltryptophan	(-) 58/74/98/116
7.15	187.0975	187.0975			$C_9H_{16}O_4$	Nonanedioic acid	(-) 97/125
9.34	327.2176	327.2176			$C_{18}H_{32}O_5$	Trihydroxyoctadecadienoic acid	(-) 171/211 /229/291
9.89	329.2333	329.2333			$C_{18}H_{34}O_5$	Trihydroxyoctadecenoic acid	(-) 171/211/229
10.82	517.3166	517.3170			$C_{30}H_{46}O_7$	Diacidic triterpenoid	(-) 377/395/421/429/ 439/455/473

Table S2. Sulfated metabolites identified by UHPLC HRMS/MS in negative ion mode in the infusion of *S. zenkeri*. Exp: experimental value. Calc: calculated value.

RT	$[M-H]^{-}$ [a] $[M-2H]^{2-}$ [b]		Molecular formula	Proposed metabolite	ESI MS/MS (-) ions (*m/z*)
	Exp m/z	***Calc m/z***			
3.73	216.9812 [a]	216.9812 [a]	$C_7H_6O_6S$	Hydroxybenzoic acid sulfate	93/97/137
5.84	457.1747 [a]	457.1748 [a]	$C_{18}H_{34}O_{11}S$	Tetrahydroxyoctadecanedioic acid sulfate	59/97/255/317/377
6.31	439.1640 [a]	439.1643 [a]	$C_{18}H_{32}O_{10}S$	Trihydroxyoctadecenedioic acid sulfate	59/97/299
6.75	427.2003 [a]	427.2007 [a]	$C_{18}H_{36}O_9S$	Tetrahydroxyoctadecanoic acid sulfate	97/347
6.81	427.2003 [a]	427.2007 [a]	$C_{18}H_{36}O_9S$	Tetrahydroxyoctadecanoic acid sulfate	97/347
6.93	505.1417 [a]<br>252.0672 [b]	505.1417 [a]<br>252.0673 [b]	$C_{18}H_{34}O_{12}S_2$	Dihydroxyoctadecanedioic acid disulfate	97/309/327/345/425
7.03	441.1797 [a]	441.1780 [a]	$C_{18}H_{34}O_{10}S$	Trihydroxyoctadecanedioic acid sulfate	97/301/361
7.12	441.1797 [a]	441.1780 [a]	$C_{18}H_{34}O_{10}S$	Trihydroxyoctadecanedioic acid sulfate	97/301/361
8.35	425.1848 [a]	425.1851 [a]	$C_{18}H_{34}O_9S$	Dihydroxyoctadecanedioic acid sulfate	97/309/327/345

Table S3. Names of sulfur-containing metabolites.

Proposed struture	Nomenclature used in the text	IUPAC nomenclature
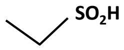	Ethane sulfinic acid	Ethanesulfinic acid
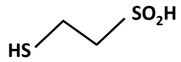	2-Sulfanylethane sulfinic acid	2-Sulfanylethane-1-sulfinic acid
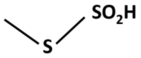	Sulfinosulfanyl methane	S-methyl hydrogen sulfurothioite
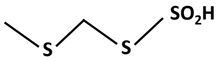	Sulfinosulfanyl 2-thiopropane	S-[(methylsulfanyl)methyl] hydrogen sulfurothioite
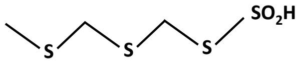	Sulfinosulfanyl 2,4 dithiopentane	S-({[(methylsulfanyl)methyl] sulfanyl} methyl) hydrogen sulfurothioite
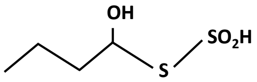	Sulfinosulfanyl hydroxybutane	S-1-hydroxybutyl hydrogen sulfurothioite
CH_3 - SO_3H	Methane sulfonic acid	Methane sulfonic acid
HOOC - CH_2- SO_3H	Sulfoacetic acid	Sulfoacetic acid
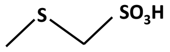	2-thiopropane sulfonic acid	(methylsulfanyl)methanesulfonic acid
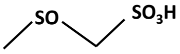	2-oxo 2-thiopropane sulfonic acid	(methylsulfinyl)methanesulfonic acid
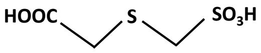	4-sulfonyl 3-thiobutanoic acid	[(sulfomethyl)sulfanyl]acetic acid
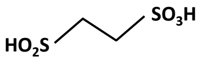	2-sulfinyl ethanesulfonic acid	2-sulfinoethane-1-sulfonic acid
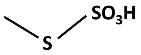	S-methyl thiosulfuric acid	S-methyl hydrogen sulfurothioate
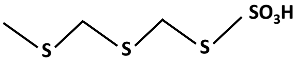	S-(2,4 dithio pentane) thiosulfuric acid	S-{[(sulfosulfanyl)methyl] sulfanyl}methyl) hydrogen sulfurothioate
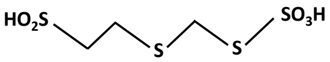	S-(2 thio butane 4-sulfinyl) thiosulfuric acid	2-{[(sulfosulfanyl)methyl] sulfanyl}ethane-1-sulfinic acid
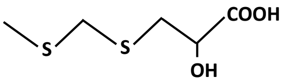	2-hydroxy 4,6-dithio heptanoic acid	2-hydroxy-3-{[(methylsulfanyl)methyl] sulfanyl} propanoic acid
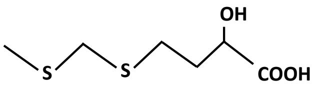	2-hydroxy 5,7-dithio octanoic acid	2-hydroxy-4-{[(methylsulfanyl)methyl] sulfanyl} butanoic acid
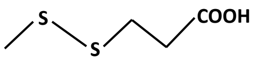	4,5-dithio hexanoic acid	3-(methyldisulfanyl)propanoic acid
